# Integrative Analysis of Long Non-coding RNAs, Messenger RNAs, and MicroRNAs Indicates the Neurodevelopmental Dysfunction in the Hippocampus of Gut Microbiota-Dysbiosis Mice

**DOI:** 10.3389/fnmol.2021.745437

**Published:** 2022-01-11

**Authors:** Lanxiang Liu, Haiyang Wang, Xueyi Chen, Yangdong Zhang, Wenxia Li, Xuechen Rao, Yiyun Liu, Libo Zhao, Juncai Pu, Siwen Gui, Deyu Yang, Liang Fang, Peng Xie

**Affiliations:** ^1^Department of Neurology, Yongchuan Hospital of Chongqing Medical University, Chongqing, China; ^2^NHC Key Laboratory of Diagnosis and Treatment on Brain Functional Diseases, The First Affiliated Hospital of Chongqing Medical University, Chongqing, China; ^3^Department of Neurology, The First Affiliated Hospital of Chongqing Medical University, Chongqing, China; ^4^College of Stomatology and Affiliated Stomatological Hospital of Chongqing Medical University, Chongqing, China; ^5^College of Biomedical Engineering, Chongqing Medical University, Chongqing, China

**Keywords:** depression, gut microbiota, lncRNAs, mRNAs, miRNAs

## Abstract

Major depressive disorder is caused by gene–environment interactions and the gut microbiota plays a pivotal role in the development of depression. However, the underlying mechanisms remain elusive. Herein, the differentially expressed hippocampal long non-coding RNAs (lncRNAs), messenger RNAs (mRNAs), and microRNAs (miRNAs) between mice inoculated with gut microbiota from major depressive disorder patients or healthy controls were detected, to identify the effects of gut microbiota-dysbiosis on gene regulation patterns at the transcriptome level, and in further to explore the microbial-regulated pathological mechanisms of depression. As a result, 200 mRNAs, 358 lncRNAs, and 4 miRNAs were differentially expressed between the two groups. Functional analysis of these differential mRNAs indicated dysregulated inflammatory response to be the primary pathological change. Intersecting these differential mRNAs with targets of differentially expressed miRNAs identified 47 intersected mRNAs, which were mainly related to neurodevelopment. Additionally, a microbial-regulated lncRNA–miRNA–mRNA network based on RNA–RNA interactions was constructed. Subsequently, according to the competitive endogenous RNAs (ceRNA) hypothesis and the biological functions of these intersected genes, two neurodevelopmental ceRNA sub-networks implicating in depression were identified, one including two lncRNAs (4930417H01Rik and AI480526), one miRNA (mmu-miR-883b-3p) and two mRNAs (*Adcy1* and *Nr4a2*), and the other including six lncRNAs (5930412G12Rik, 6430628N08Rik, A530013C23Rik, A930007I19Rik, Gm15489, and Gm16251), one miRNA (mmu-miR-377-3p) and three mRNAs (*Six4*, *Stx16*, and *Ube3a*), and these molecules could be recognized as potential genetic and epigenetic biomarkers in microbial-associated depression. This study provides new understanding of the pathogenesis of depression induced by gut microbiota-dysbiosis and may act as a theoretical basis for the development of gut microbiota-based antidepressants.

## Introduction

Major depressive disorder (MDD) is a heterogeneous and multifactorial psychiatric disorder. Globally, more than 350 million people suffer from depression and the lifetime prevalence of MDD is 6.8% (GBD 2017 Disease and Injury Incidence and Prevalence Collaborators, 2018). Gene–environment interaction plays a crucial role in the etiology of MDD (Assary et al., 2018). The gut microbiota, a community of microorganisms in the gastrointestinal tract, is a pivotal environmental factor that is recognized to play an important role in regulating human health and disease through microbiota–host bidirectional communication (Lynch and Pedersen, 2016). In genetically susceptible individuals, the gut microbiota may interact with genetic factors to co-regulate the host’s disease symptoms. Alternatively, a “pathogenic” microbiota may be sufficient to trigger a psychiatric condition, such as depression, even without a genetic risk.

Abundant preclinical and clinical evidence indicates moderating effects of the gut microbiota on the onset of depression. Significantly, disturbances in the gut microbiota, characterized by alterations in the relative abundances of the phyla *Actinobacteria*, *Bacteroidetes*, *Firmicutes*, and *Proteobacteria*, were identified in patients with MDD (Jiang et al., 2015; Zheng et al., 2016; Simpson et al., 2021). Moreover, transplant of fecal microbiota from patients with MDD into microbiota-deficient rodents promoted depressive-like behaviors indicating a causal role of gut microbiota dysbiosis in depression (Kelly et al., 2016; Zheng et al., 2016). A key question is, therefore, how do these “pathogenic” microbiota trigger the development of depression? Gut microbiota can regulate depression via the microbiota-gut-brain axis (Foster and McVey Neufeld, 2013). Previously, disruption to mitochondria-mediated biological processes, the MAPK pathway and the CAMKII-CREB pathway, and the hypothalamic-pituitary-adrenal (HPA) axis were identified in gut microbiota-dysbiosis depressed mice (Li et al., 2018; Liu et al., 2020a; Wang et al., 2020). Many pathways in the gut-brain axis, from intricate neuronal pathways to subtle small molecule messaging systems, are involved in the mechanisms by which gut microbiota dysbiosis causes depression-related brain dysfunction and behavioral changes (Cryan et al., 2019). However, the exact mechanisms are complex and remain incompletely understood.

Psychiatric disorders are characterized by transcriptional dysregulation (Gandal et al., 2018; Egervari et al., 2019), and transcriptional signatures of depression have been described (Li et al., 2021; Seney et al., 2021). MicroRNAs (miRNAs) are small non-coding RNAs of 18–25 nucleotides that can suppress gene expression by degrading and/or repressing translation of target mRNAs after binding to complementary sequences in the 3′-untranslated region (UTR) (De Martinis et al., 2020). Long non-coding RNAs (lncRNAs) are more than 200 nucleotides in length and outnumber mRNAs by 3–100 times. However, the function of most lncRNAs is still not clear. lncRNAs can regulate protein-coding genes at various levels, e.g., at epigenetic, transcriptional and post-transcriptional levels (Yao et al., 2019). lncRNAs can act as competitive endogenous RNAs (ceRNAs) that sponge miRNAs through microRNA response elements (Salmena et al., 2011), thereby regulating gene expression of target mRNAs. The absence of gut microbiota can cause changes to the expression of hippocampal mRNAs, miRNAs and lncRNAs (Moloney et al., 2017; Liu et al., 2020b), indicating a regulatory role of gut microbiota in transcriptional activity. However, the microbial-regulated lncRNA–miRNA–mRNA ceRNA network has not been investigated with respect to gut microbiota-dysbiosis-induced depression.

To comprehensively understand microbiota-regulated transcriptional networks in depression, the expression profiling of lncRNA, mRNA, and miRNA in the hippocampus of mice that had been inoculated with “depression microbiota” (microbiota from fecal samples of MDD patients) or “healthy microbiota” (microbiota from fecal samples of healthy controls) were analyzed, and the disturbed biological functions was explored to uncover the pathological mechanisms of gut microbiota-dysbiosis-regulated depression. Finally, a lncRNA–miRNA–mRNA ceRNA network was constructed based on the RNA-RNA interactions.

## Materials and Methods

### Animals

Eighteen male germ-free Kunming mice (8-week-old, 30–40 g) were used in this study. During the experiment, all mice were kept under standard environmental conditions with a 12 h light/dark cycle, a temperature of 22–24°C and humidity of 45–55%. The germ-free status of mice was verified to meet the Chinese Laboratory Animal Microbiological Standards and Monitoring (GB 14922.2-2011) via testing the feces and skin. This experiment was performed in accordance with NIH Guidelines (No. 8023, revised 1978) and approved by the Ethics Committee of Chongqing Medical University.

### Fecal Microbiota Transplantation

As previously described (Liu et al., 2020a), after collecting the fecal samples from MDD patients and healthy controls, 100 milligrams of feces from each sample were suspended in 1.5 ml 0.9% sterile saline, and equal volumes of suspensions were mixed to generate a “depression microbiota” pool and a “healthy microbiota” pool. Germ-free mice (8-week-old) were randomly inoculated with 200 μl pooled samples by gavage to generate “depression microbiota” and “healthy microbiota” recipient mice. To avoid interplay of gut microbiota, mice in the two groups were kept in a separate gnotobiotic isolator. Two weeks is sufficient for successful colonization after fecal microbiota transplantation and is the benchmark time point for assessing disease phenotypes (Turnbaugh et al., 2006; Koren et al., 2012). Therefore, depression-related changes in mice were evaluated 2 weeks after colonization in present study.

### Sample Collection

After anesthesia with 10% chloral hydrate (200 mg/kg, i.p.), mice were killed and the entire hippocampus, a key brain area in the neural circuitry of depression (Mayberg, 2009), was collected, rapidly frozen in liquid nitrogen, and stored at –80°C until microarray analysis. Hippocampal tissues from three randomly chosen experimental mice were mixed as a sample pool, generating three sample pools per group.

### Microarray Analysis

#### Long Non-coding RNAs and Messenger RNAs Profiling

Total RNA was extracted using TRIzol^®^ Reagent (Invitrogen, United States) and quantified using a NanoDrop ND-1000. Purified mRNA was obtained after removing ribosomal RNA using an mRNA-ONLY™ Eukaryotic mRNA Isolation Kit (Epicentre, United States). After labeling with a Quick Amp Labeling Kit, One-Color (Agilent, United States), the prepared RNA sample was amplified and transcribed into fluorescent cRNA using a random priming method. The labeled cRNAs were purified using an RNeasy Mini Kit (Qiagen, Germany) and hybridized onto a Mouse lncRNA Array v2.0 (8 × 60K, Arraystar, United States). Arrays were scanned with the Agilent Scanner G2505C and the acquired images were analyzed using Agilent Feature Extraction software (v 11.0.1.1). The raw data was normalized using the GeneSpring GX v12.0 software package (Agilent, United States). lncRNAs and mRNAs for which at least three out of six samples had Present or Marginal flags (“All Targets Value”) were used for further analysis. Differentially expressed lncRNAs (DELs) and differentially expressed mRNAs (DEGs) between “depression microbiota” and “healthy microbiota” recipient mice with an absolute fold-change ≥ 1.5 and a false discovery rate (FDR) < 0.05 were selected for further functional analysis. Hierarchical clustering was performed to show distinguishable DEL and DEG expression patterns among samples.

#### MicroRNAs Profiling

Total RNA was harvested using TRIzol^®^ Reagent (Invitrogen, United States) and an miRNeasy mini kit (Qiagen, Germany) according to manufacturers’ instructions. The quality and quantity of RNA was measured using a NanoDrop ND-1000 and RNA integrity was determined by gel electrophoresis. Samples were labeled using the miRCURY™ Hy3™/Hy5™ Power labeling kit (Exiqon, Denmark) and hybridized on a 7th generation miRCURY™ LNA Array (v.18.0) (Exiqon, Denmark). An Axon GenePix 4000B was used to scan the slides and the images were processed using GenePix Pro 6.0 software (Axon). The average was calculated for replicated miRNAs. miRNAs with an intensity ≥ 30 were chosen. After Median normalization, differentially expressed miRNAs (DEMs) were identified based on the thresholds of an absolute fold-change ≥ 1.5 and a FDR < 0.05.

### Prediction of Differentially Expressed MicroRNAs and Differentially Expressed Long Non-coding RNAs Targets

Target mRNAs of DEMs were first predicted using TargetScanMouse 7.2 (Agarwal et al., 2015), miRDB (Chen and Wang, 2020), DIANA-TarBase v8 (Karagkouni et al., 2018), and miRTarBase 8.0 (Chou et al., 2018) online tools, and by microRNA Target Filter analysis based on the Ingenuity Pathway Analysis (IPA) database. In addition, target lncRNAs of DEMs were analyzed using ENCORI-starBase v2.0 (Li et al., 2014) and DIANA-LncBase v2 (Paraskevopoulou et al., 2016) tools. Finally, the target mRNAs of DELs were defined as *cis*-regulated genes located within 300 kb upstream or downstream of the genomic location of the DELs.

### Functional Pathway and Network Enrichment Analysis

Gene ontology (GO) analysis was applied to DEGs and the intersected mRNA targets of DEMs to determine biological process, cellular component and molecular function GO terms using OmicsBean online software^1^ with default parameters. To reveal potential pathological mechanisms of gut microbiota-dysbiosis induced depression, enrichment analysis to identify functional pathways and networks were performed through uploading the gene lists (gene symbols) with the corresponding fold changes and *p*-values onto the IPA software^2^, and the analysis parameters were default. Moreover, gene-gene interactions were analyzed based on the STRING database^3^, and an interaction with a confidence score > 0.4 was included in further network analysis. Then, the gene interaction networks were visualized using Cytoscape software (v3.7.2) (Su et al., 2014) on the basis of gene-gene interactions. Highly connected clusters were identified using the MCODE plug-in (Bader and Hogue, 2003) and the hub gene was analyzed using the NetworkAnalyzer plug-in (Doncheva et al., 2012).

### Construction of the lncRNA–miRNA–mRNA Competitive Endogenous RNAs Network

To identify the key genes in microbial-associated depression, intersection analysis of the DEGs and the target genes of DEMs was performed, and the intersected mRNAs sets were used to analyze the mRNA–mRNA interactions, as well as miRNA–mRNA regulatory pairs. In addition, the DELs were also intersected with the target lncRNAs of DEMs, and the intersected lncRNAs were selected to generate miRNA–lncRNA pairs. Subsequently, the lncRNA–miRNA–mRNA network was constructed on the basis of mRNA–mRNA interactions, miRNA–mRNA regulatory correlations, and miRNA–lncRNA relationships. According to the ceRNAs hypothesis, the DEMs and their negatively regulated DEGs and DELs were selected to generate the lncRNA–miRNA–mRNA ceRNA sub-networks. The final networks were visualized using Cytoscape software (v3.7.2).

## Results

### Quality Assessment of RNA Data

Quality of RNA data was assessed after low intensity filtering. Box-plots of the lncRNA and mRNA indicated the distributions of the normalized intensities among all tested samples were nearly the same (Supplementary Figures 1A,B). Scatter-plots indicated the variation and reproducibility of the lncRNA and mRNA expression between the two compared groups (Supplementary Figures 1C,D). A box-plot of the miRNA is shown in Supplementary Figure 2 to visualize the distribution of each sample, and a scatter-plot to assess the correlation among replicate experiments is shown in Supplementary Figure 3. Microarray-based analysis of hippocampal tissues from gut microbiota-dysbiosis mice identified the levels of lncRNAs, mRNAs, and miRNAs.

### Functional Analysis of DEGs in Response to Gut Microbiota-Dysbiosis

**FIGURE 1:**
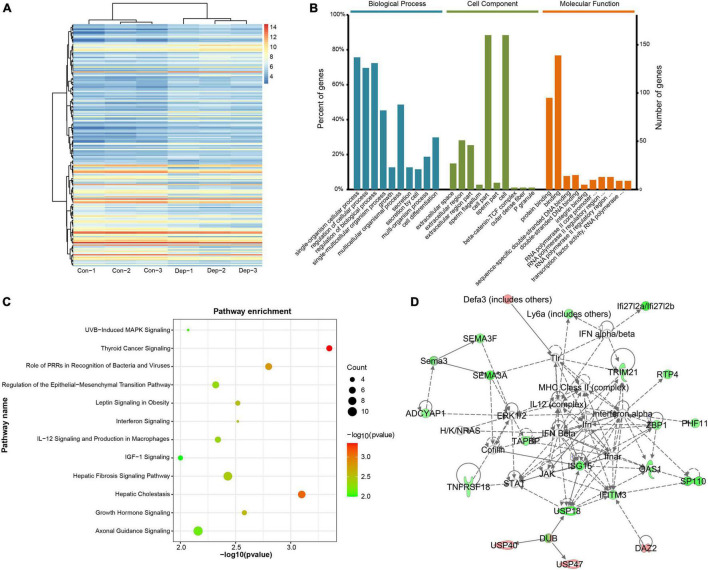
Identification and functional analysis of differentially expressed mRNAs (DEGs) in gut microbiota-dysbiosis mice. **(A)** Hierarchical clustering of DEGs. **(B)** Gene ontology (GO) annotations of DEGs. **(C)** Canonical pathway enrichment for DEGs analyzed by Ingenuity Pathway Analysis (IPA) software. **(D)** Functional network analysis for DEGs revealed by IPA software.

In total, 19,775 mRNAs in the hippocampus of gut microbiota-dysbiosis mice were quantified. Based on the aforementioned screening criteria for differentially expressed RNAs, 200 DEGs were identified between the two groups (Supplementary Table 1). Of these, 112 were up-regulated and 88 were down-regulated in “depression microbiota” inoculated mice compared with “healthy microbiota” inoculated mice (Figure 1A). DEGs ranged in length from 427 to 15,363 nucleotides, with the average being 2922 nucleotides. GO annotations were performed to explore the functions of DEGs and DEGs, and several relevant GO terms were enriched (Figure 1B). Single-organism cellular process was the dominant GO term in biological process annotations (Supplementary Table 2). For the cellular component category, the DEGs were mainly annotated to extracellular space (Supplementary Table 3), and most DEGs under molecular function terms were annotated to binding, especially protein binding (Supplementary Table 4).

**FIGURE 2:**
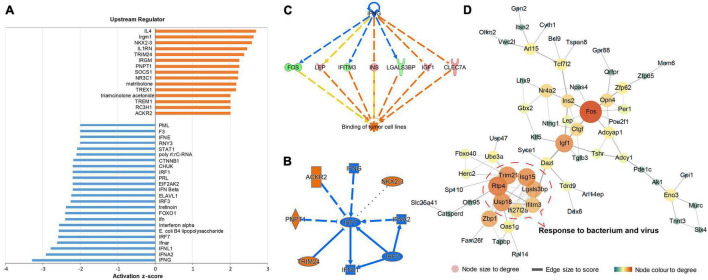
Upstream regulator prediction and protein–protein interaction (PPI) analysis of DEGs in gut microbiota-dysbiosis mice. **(A)** Histogram showing the inhibited and activated upstream regulators of DEGs. **(B)** Graphical summary of the upstream regulators. **(C)** The highest-ranked upstream regulator, IFNG, and its downstream DEGs. **(D)** PPI network analysis of DEGs.

To explore the biological processes affected by gut microbiota-dysbiosis in mice, functional pathway and network analysis were performed. Significant canonical pathways, displayed in Figure 1C, were disrupted in several biological functions related to the inflammatory response, including the role of pattern recognition receptors in the recognition of bacteria and viruses, interferon signaling, and IL-12 signaling and production in macrophages. DEGs were most enriched in axonal guidance signaling function. Network analysis indicated that antimicrobial response, inflammatory response, cellular assembly and organization were the most significantly altered biological functions (Figure 1D). Consistently, upstream regulator analysis identified the inhibition of interferons, e.g., interferon gamma (IFNG), IFNA2, IFNL1, IRF3, and IRF7, and the activation of ACKR2, PNPT1, TRIM24, and NKX2-3 (Figures 2A,B), indicating perturbations of the inflammatory response in gut microbiota-dysbiosis-induced depression mice. Of the seven DEGs that led to the prediction of IFNG as the highest-ranked upstream regulator, three (*Fos*, *Lgals3bp*, and *Ifitm3*) were down-regulated and four (*Lep*, *Ins*, *Igf1*, and *Clec7a*) were up-regulated (Figure 2C).

Identification of gene–gene interactions was performed to further explore the pathological mechanisms of gut microbiota-dysbiosis induced depression. Our DEGs dataset produced a gene–gene network with a highly connected cluster of nodes, including *Ifi27l2a*, *Ifitm3*, *Isg15*, *Lgals3bp*, *Usp18*, *Trim21*, and *Rtp4*. The proteins encoded by these genes have synergistic functions in response to bacteria and viruses (Figure 2D). In addition, *Fos*, with the highest degree of interaction, was recognized as a hub gene in this gene-gene network. Interestingly, *Fos* is a transcription factor and its mRNA level can act as an index of neuronal activity. Taken together, the inhibition and activation of upstream cytokines, as well changes in the expression of related genes, indicated that dysregulation in the inflammatory response caused by gut microbiota-dysbiosis may lead to decreased neuronal activity, characterized by the down-regulation of *Fos* mRNA.

### Functional Analysis of Differentially Expressed Long Non-coding RNAs in Response to Gut Microbiota-Dysbiosis

**FIGURE 3:**
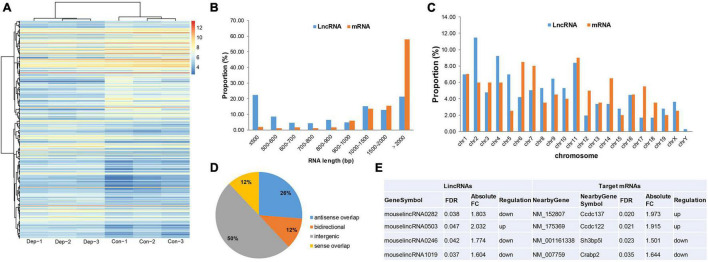
Identification and functional analysis of differentially expressed lncRNAs (DELs) in gut microbiota-dysbiosis mice. **(A)** Hierarchical clustering of DELs. **(B)** Comparison of RNA length for DELs versus DEGs. **(C)** Comparison of chromosome distribution for DELs versus DEGs. **(D)** Classification of DELs based on the relationship between lncRNAs and their associated protein-coding genes. **(E)** Intergenic lncRNAs and their *cis*-regulated nearby genes. FDR, false discovery rate; FC, fold change.

Microarray-based analysis identified 26,494 lncRNAs and 358 DELs (195 up-regulated, 163 down-regulated) between “depression microbiota” and “healthy microbiota” inoculated mice (Figure 3A and Supplementary Table 5). When DELs were compared with DEGs, half of the DELs were less than 1000 bp in length, while more than half of the DEGs were more than 2000 bp in length (Figure 3B). The chromosome distribution of DELs and DEGs is displayed in Figure 3C. According to the relationship between DELs and their associated protein-coding genes, DELs detected by Arraystar Microarray were classified as intergenic (50%), antisense overlap (26%), sense overlap (12%), and bidirectional (12%) lncRNAs (Figure 3D).

lncRNA subgroup analysis was performed to identify putative functional relationships between DELs and their associated protein-coding genes. Intergenic lncRNAs (lincRNAs) were the most abundant subgroup of lncRNAs, so they were used for further functional analysis. To uncover potential functions of these differentially expressed lincRNAs, *cis*-regulated, nearby genes, located within 300 kb, were predicted. After intersecting with DEGs, four target genes were identified for four lincRNAs (Figure 3E). *Sh3bp5l* was down-regulated in gut microbiota-dysbiosis-induced depression mice. SH3BP5L is an SH3 domain-binding protein that plays important roles in regulating proteins or signaling pathways associated with development (Hu et al., 2008). In addition, *Crabp2* was also down-regulated, and the protein it encodes functions in transporting retinoic acid from the cytosol to the nucleus. However, the functions of *Ccdc137* and *Ccdc122* remain largely unknown.

### Functional Analysis of Differentially Expressed MicroRNAs in Response to Gut Microbiota-Dysbiosis

**FIGURE 4:**
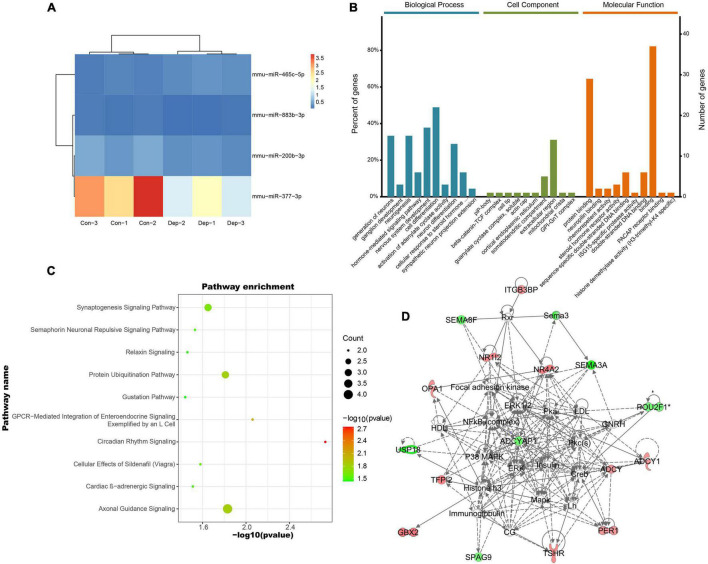
Identification and functional analysis of differentially expressed miRNAs (DEMs) in gut microbiota-dysbiosis mice. **(A)** Hierarchical clustering of DEMs. **(B)** GO annotations of the intersected DEM targets. **(C)** Canonical pathway enrichment for the 47 intersected DEM targets analyzed using IPA software. **(D)** Functional network analysis for the 47 intersected DEM targets revealed by IPA software.

A total of 3552 miRNAs expressed in the hippocampus of gut microbiota-dysbiosis mice were identified. Screening of these miRNAs for differential expression revealed three DEMs, mmu-miR-465c-5p, mmu-miR-200b-3p, and mmu-miR-883b-3p (Figure 4A). mmu-miR-377-3p with a *p*-value less than 0.05 has a significant trend of decrease after FDR correction in “depression microbiota” inoculated mice, and was also included for further analysis. miRNAs can regulate gene expression by binding to the complementary sequences of their targets; therefore, putative biological functions of these four DEMs can be proposed by the functions of their target genes. Using online prediction tools, 4632 target mRNAs were identified for these four DEMs. After intersecting the target genes with the DEGs, 47 intersecting mRNAs were obtained and were used for further functional analysis. The miRNA-mRNA interaction pairs are shown in Table 1.

**TABLE 1:** Differentially expressed miRNAs and their differentially expressed mRNA targets.

miRNAs					Target mRNAs				
ID	Name	FC^a^	FDR^b^	Regulation	Seqname	GeneSymbol	FC (abs)^c^	FDR^b^	Regulation
42519	mmu-miR-465c-5p	1.532561563	0.049692	Up	NM_001033407	Gm815	1.521011	0.04507730	Down
					NM_010738	Ly6a	1.621894	0.03735421	Down
					NM_001163350	Ntng1	1.795526	0.04875637	Down
					NM_001025568	Pde1c	1.544196	0.04748477	Down
					NM_198932	Pou2f1	1.5370071	0.04118802	Down
					NM_009152	Sema3a	2.5020425	0.04096039	Down
					NM_177164	Vwc2l	1.8152527	0.02411733	Down
					NM_145575	Cald1	1.602462	0.04895628	Up
					NM_001037321	Fbxo40	1.5813568	0.02854253	Up
					NM_010262	Gbx2	2.0050368	0.04074322	Up
					NM_010418	Herc2	1.6062031	0.04839108	Up
					NM_026348	Itgb3bp	1.5665871	0.00909908	Up
					NM_001098404	Nr1i2	1.7842534	0.04952551	Up
					NM_133752	Opa1	1.6633599	0.03013054	Up
					NM_001003717	Osbpl8	1.6370533	0.04842034	Up
					NM_028748	Paqr5	1.7612659	0.02971225	Up
					NM_001159367	Per1	1.567625	0.02170788	Up
					NM_011137	Pou2f1	1.5176835	0.02779596	Up
					NM_178227	Scn3b	1.6376927	0.02688412	Up
					NM_001113404	Tshr	1.7740262	0.03473434	Up
147186	mmu-miR-200b-3p	0.658253975	0.041228	down	NM_009649	Akap2	1.522913	0.04842372	Up
					NM_172595	Arl15	1.5095077	0.04860469	Up
					NM_029933	Bcl9	1.5354732	0.02891231	Up
					NM_001081225	Fam178a	1.6083025	0.04959859	Up
					NM_030201	Hspa13	3.1505609	0.01178443	Up
					NM_011365	Itsn2	1.7630032	0.03734254	Up
					NM_178227	Scn3b	1.6376927	0.02688412	Up
					NM_173010	Ube3a	1.6787672	0.01200062	Up
					NM_001109691	Phf21a	1.859211	0.03746215	Down
					NM_011081	Piga	1.6523745	0.04463066	Down
					NM_009152	Sema3a	2.5020425	0.04096039	Down
					NM_011349	Sema3f	1.7213542	0.04393336	Down
					NM_001199205	Spag9	1.517349	0.04762527	Down
					NM_029056	Tdrd9	1.5761601	0.04880729	Down
					NM_001101640	Tmem207	1.8919897	0.04882137	Down
					NM_011909	Usp18	2.5167851	0.02862872	Down
42883	mmu-miR-883b-3p	0.569539569	0.039109	Down	NM_009622	Adcy1	1.580024	0.03985197	Up
					NM_172595	Arl15	1.5095077	0.04860469	Up
					NM_001163333	Cttnbp2nl	1.7116936	0.01972822	Up
					NM_152895	Kdm5b	1.687644	0.04898798	Up
					NM_001139509	Nr4a2	1.9803841	0.04820689	Up
					NM_133752	Opa1	1.6633599	0.03013054	Up
					NM_009364	Tfpi2	1.9140846	0.04680254	Up
					NM_009625	Adcyap1	1.6437125	0.03973875	Down
					NM_009152	Sema3a	2.5020425	0.04096039	Down
					NM_001039967	Zfp869	1.7256061	0.02157675	Down
11091	mmu-miR-377-3p	0.471786427	0.066332*	down	NM_021483	Pex5l	1.618897	0.03024903	Up
					NM_011382	Six4	1.510553	0.04898937	Up
					NM_177829	Spink10	1.535757	0.02555243	Up
					NM_001102425	Stx16	1.6225739	0.04827980	Up
					NM_173010	Ube3a	1.6787672	0.01200062	Up
					NM_019626	Cbln1	2.1865587	0.03002855	Down
					NM_001164518	Iglon5	1.9751669	0.02888774	Down
					NM_001025568	Pde1c	1.544196	0.04748477	Down
					NM_001109691	Phf21a	1.859211	0.03746215	Down

*^a^Fold change = the ratio of normalized intensities between two conditions (use normalized data, ratio scale). Normalized Data = (Foreground–Background)/median.*

*^b^False discovery rate that was calculated by Benjamini–Hochberg method.*

*^c^Absolute fold change = the absolute ratio (no log scale) of normalized intensities between two conditions.*

*This miRNA has a p-value < 0.05.

The enriched GO terms of these intersected targets are displayed in Figure 4B. Significantly, the generation of neurons, ganglion development, and neurogenesis were the primarily enriched biological process annotations (Supplementary Table 6). Meanwhile, the somatodendritic compartment and protein binding were the most significant terms in the cellular component and molecular function categories, respectively (Supplementary Tables 7, 8). Functional pathway analysis found that most target genes were enriched in axonal guidance signaling (*Herc2*, *Ntng1*, *Sema3a*, and *Sema3f*) and synaptogenesis signaling (*Adcy1*, *Itsn2*, and *Stx16*) (Figure 4C). Cellular assembly and organization, cellular compromise, nervous system development and function were the top-ranked biological functions (Figure 4D). Thus, the biological functions of these DEMs were mainly related to neurodevelopment.

### Competitive Endogenous RNAs Regulatory Network in Response to Gut Microbiota-Dysbiosis

**FIGURE 5:**
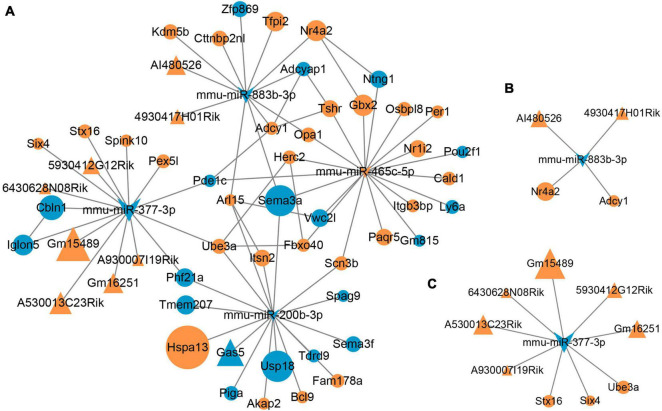
Microbial-regulated lncRNA–miRNA–mRNA network construction. **(A)** The lncRNA–miRNA–mRNA regulatory network constructed based on the RNA–RNA interactions. **(B,C)** Two ceRNA sub-networks involved in neurodevelopment were constructed according to the ceRNAs hypothesis.

The miRNA–lncRNA interactions were analyzed to identify ceRNAs that sponge miRNAs. Total of 2193 target lncRNAs for these four DEMs were predicted, and nine lncRNAs survived after intersecting the target lncRNAs with the DELs. The resulting miRNA-lncRNA interaction pairs are shown in Table 2. Combining with the miRNA–mRNA interactions mentioned above, as well as the gene-gene interactions for the 47 intersected mRNAs, a lncRNA–miRNA–mRNA regulatory network was constructed (Figure 5A). According to the ceRNA hypothesis and the biological process annotations for these 47 intersecting genes, two sub-transcriptional ceRNA networks were re-constructed, one including two lncRNAs (4930417H01Rik and AI480526), one miRNA (mmu-miR-883b-3p) and two mRNAs (*Adcy1* and *Nr4a2*) (Figure 5B), and the other including six lncRNAs (5930412G12Rik, 6430628N08Rik, A530013C23Rik, A930007I19Rik, Gm15489, and Gm16251), one miRNA (mmu-miR-377-3p) and three mRNAs (*Six4*, *Stx16*, and *Ube3a*) (Figure 5C). These two ceRNA sub-networks are indicated to play important roles in neurodevelopment.

**TABLE 2:** Differentially expressed miRNAs and their differentially expressed lncRNAs targets.

ID	Name	FC^a^	FDR^b^	Regulation	Seqname	GeneSymbol	FDR^b^	FC(abs)^c^	Regulation
147186	mmu-miR-200b-3p	0.658254	0.041228	Down	ENSMUST00000159119	Gas5	0.04911062	2.3057933	Down
42883	mmu-miR-883b-3p	0.56954	0.039109	Down	ENSMUST00000148977	4930417H01Rik	0.04292526	1.6698261	Up
					ENSMUST00000160227	AI480526	0.03374033	1.9144645	Up
11091	mmu-miR-377-3p	0.471786	0.066332*	Down	ENSMUST00000126472	5930412G12Rik	0.03637584	1.8078425	Up
					ENSMUST00000165938	6430628N08Rik	0.04965351	1.5305915	Up
					NR_015500	A530013C23Rik	0.04294613	2.0565884	Up
					ENSMUST00000155013	A930007I19Rik	0.04227756	1.5199345	Up
					ENSMUST00000132811	Gm15489	0.04533299	2.6704226	Up
					ENSMUST00000162649	Gm16251	0.04966378	1.9383893	Up

*^a^Fold change = the ratio of normalized intensities between two conditions (use normalized data, ratio scale). Normalized Data = (Foreground–Background)/median.*

*^b^False discovery rate that was calculated by Benjamini–Hochberg method.*

*^c^Absolute fold change = the absolute ratio (no log scale) of normalized intensities between two conditions.*

*This miRNA has a p-value < 0.05.

## Discussion

The composition of the gut microbiota in MDD is inconsistent across studies; however, higher abundance of *Actinobacteria* and *Eggerthella* and lower abundance of *Bacteroidetes*, *Prevotellaceae*, *Coprococcus*, *Faecalibacterium*, and *Sutterella* are the most consistent findings in patients with MDD compared with healthy controls (Simpson et al., 2021). Previously reports have found that germ-free mice inoculated with fecal microbiota from patients with MDD exhibited typical depressive-like behaviors, characterized by an increase in immobility time in the forced swimming and tail suspension tests compared with germ-free mice inoculated with fecal microbiota from healthy controls (Zheng et al., 2016; Liu et al., 2020a). These behavioral phenotypes may have been caused by mono-colonization or synergism of multiple bacterial species; however, the potential mechanisms by which gut microbiota-dysbiosis regulate depressive-like behaviors were complex and still unclear. In the present study, microarrays were used to profile transcript changes in gut microbiota-dysbiosis mice. Integrated analysis found that dysregulation of the inflammatory response and neurodevelopment were the primary pathological changes. In addition, two neurodevelopmental-associated lncRNA–miRNA–mRNA ceRNA regulatory network were constructed in gut microbiota-dysbiosis-induced depression, one including two lncRNAs (4930417H01Rik and AI480526), one miRNA (mmu-miR-883b-3p) and two mRNAs (*Adcy1* and *Nr4a2*), and the other including six lncRNAs (5930412G12Rik, 6430628N08Rik, A530013C23Rik, A930007I19Rik, Gm15489, and Gm16251), one miRNA (mmu-miR-377-3p) and three mRNAs (*Six4*, *Stx16*, and *Ube3a*), and these molecules could be recognized as potential genetic and epigenetic biomarkers in microbial-associated depression.

According to the hygiene hypothesis, the development and balance of the immune system are closely related to gut microbiota colonization (Bach, 2018). Under conditions of gut microbiota-dysbiosis, toxins and harmful bacteria can trigger an imbalanced inflammatory response that can lead to autoimmune diseases (Pickard et al., 2017). MDD was characterized to have a lower abundance of anti-inflammatory bacteria (e.g., *Coprococcus* and *Faecalibacterium*) and a higher relative abundance of pro-inflammatory species (e.g., *Eggerthella*) (Simpson et al., 2021). In addition to the effects of gut microbiota *per se*, imbalanced inflammatory responses are widely recognized to contribute to the pathogenesis of depression (Gałecki and Talarowska, 2018). In patients with depression, the levels of CRP, IL-6, IL-12, and TNF-α are significantly elevated, indicating a pro-inflammatory state (Osimo et al., 2020). Clinical studies have shown anti-inflammatory agents, in particular cytokine-inhibitors and non-steroidal anti-inflammatory drugs (NSAIDs), to have antidepressant effects either as an add-on strategy or as monotherapy (Kohler et al., 2016), indicating an etiological role of inflammation in depression. In the present study, several biological functions related to the inflammatory response were dysregulated, such as the role of pattern recognition receptors in the recognition of bacteria and viruses, interferon signaling, and IL-12 signaling and production in macrophages. Previous study has indicated that transplanting the fecal microbiota from chronic unpredictable mild stress mice caused depressive-like behaviors in recipient mice via the gut microbiota-inflammation-brain axis (Li et al., 2019). Dysregulation of the downstream inflammatory response leads to the inhibition or activation of the corresponding upstream regulators, e.g., the inhibition of IFNG, IFNA2, IFNL1, IRF3, and IRF7, and the activation of ACKR2, PNPT1, TRIM24, and NKX2-3. As the highest-ranked upstream regulator, IFNG was predicted to be inhibited, and its downstream target, *Fos*, an index of neuronal activity, was down-regulated in gut microbiota-dysbiosis mice. IFNG has been proved to play a role in the development of depressive-like behaviors and its related hippocampal neurogenesis through regulating the function of microglia (Zhang et al., 2020). Neuroinflammatory-mediated mechanisms can influence neuronal activity as well as synaptic plasticity and have been implicated in the neurobiology of depression (Wohleb et al., 2016). Thus, it is inferred that dysregulation of the inflammatory response caused by gut microbiota-dysbiosis may lead to decreased neuronal activity in depression, characterized by the down-regulation of *Fos* mRNA.

Brain development occurs in parallel with gut microbiota development during early postnatal life (Tognini, 2017; Wang et al., 2018), indicating an essential role of gut microbiota in neurodevelopment, including neurogenesis, myelination, microglia maturation, and formation of the blood-brain barrier (Sharon et al., 2016). Imbalance in the relative abundance of specific “pathogenic” bacteria in the gut microbiota is strongly associated with the onset of psychiatric disorders, e.g., depression, possibly because of altered neurodevelopment (Lima-Ojeda et al., 2017; Settanni et al., 2021). Both germ-free mice and antibiotic-treated specific pathogen free mice show alterations in hippocampal neurogenesis (Möhle et al., 2016), as well as in cortical myelination and myelin plasticity (Hoban et al., 2016). Deficient hippocampal neurogenesis is implicated in the pathogenesis of depression (Snyder et al., 2011), while increasing hippocampal neurogenesis is sufficient to recover depressive-like behaviors in mice (Hill et al., 2015). In this study, GO analysis showed that the intersected DEGs regulated by DEMs were mainly enriched in neurodevelopment, in particular axonal guidance signaling (*Herc2*, *Ntng1*, *Sema3a*, and *Sema3f*) and synaptogenesis (*Adcy1*, *Itsn2*, and *Stx16*), as well as neuron generation, ganglion development and neurogenesis. According to the negative regulatory relationships between mRNAs and miRNAs, it is inferred that the microbial-regulated pairs of mmu-miR-465c-5p-*Ntng1*, mmu-miR-465c-5p-*Sema3a*, mmu-miR-883b-3p-*Adcy1*, mmu-miR-200b-3p-*Itsn2*, and mmu-miR-377-3p-*Stx16* play important roles in neurodevelopment. Interestingly, miR-200 microRNA family has been recently identified as an important regulator of gliogenesis and neurogenesis and of adult neural homeostasis in the central nervous system of rodents (Trümbach and Prakash, 2015), and the expression levels of miR-200 family were elevated in mature and differentiated neurons (Jauhari and Yadav, 2019). The downregulation of mmu-miR-200b-3p in present study suggested the impairment of neurodevelopment in gut microbiota-dysbiosis induced depression mice. However, the exact effects of new genetic biomarkers, i.e., mmu-miR-465c-5p, mmu-miR-883b-3p, and mmu-miR-377-3p, on neurodevelopment needed to be further warranted. Consistently, our previous phosphoproteomics study indicated that axon guidance was the primary functional change shared among gut microbiota-dysbiosis-induced depression mice, stress-induced depression rats and MDD postmortem brains (Wang et al., 2020). Taken together, it is suggested that alterations in neurodevelopment, such as axonal guidance, neurogenesis and myelination, underlie the pathological mechanisms by which gut microbiota-dysbiosis induces depression in mice.

It is speculated that miRNAs regulate approximately one third of human genes. miRNAs can suppress gene expression through mRNA translational repression, degradation, or both. In addition to miRNAs, lncRNAs are also emerging as important regulators of gene expression at epigenetic, transcriptional and post-transcriptional levels (Yao et al., 2019). Based on the ceRNA hypothesis, in which lncRNAs act as ceRNAs that sponge miRNAs via microRNA response elements to regulate mRNA, two microbial-regulated lncRNA–miRNA–mRNA ceRNA networks that play important roles in neurodevelopment in depression were constructed in present study. From these two sub-networks, these eight novel lncRNAs (4930417H01Rik, AI480526, 5930412G12Rik, 6430628N08Rik, A530013C23Rik, A930007I19Rik, Gm15489, and Gm16251), two miRNAs (mmu-miR-883b-3p and mmu-miR-377-3p) and five mRNAs (*Adcy1*, *Nr4a2*, *Six4*, *Stx16*, and *Ube3a*) were speculated as key molecules in gut microbiota-dysbiosis-induced depression, and these molecules could be recognized as potential genetic and epigenetic biomarkers in microbial-associated depression. miR-377-3p functions in regulating the inflammatory response, cell proliferation and apoptosis rate (Sun et al., 2021). Previously, dysregulated expression of *Adcy1* mRNA was identified in the hippocampus and prefrontal cortex of restraint stress induced depression mice, and suggested *Adcy1* as a potential biomarkers in depression, and may act as a target in treatment of depression (Yang et al., 2020). *Nr4a2* gene polymorphisms were found to be associated with several symptoms of MDD, and may be a predictor of antidepressant efficacy (Song et al., 2021). Whereas, further evidences were still required to verify the potential roles of the remaining molecules as genetic and epigenetic biomarkers in depression.

Integrated analysis of lncRNAs, miRNAs, and mRNAs enables us to comprehensively screen the potential genetic and epigenetic biomarkers in depression at the transcriptional level and clarify their regulatory roles in the microbiota-gut-brain axis, which will be essential to explore the diagnostic biomarkers and therapeutic targets for depression, especially in the gut microbiota-dysbiosis caused depression. However, extending the present findings to the diagnosis and treatment of depression in humans is still a substantial challenge, due to the obvious biological differences between animals and humans, and the complexity, individuality, and dynamics of biological features in humans aggravate this challenge. Anyhow, the identification of these genetic and epigenetic biomarkers promotes the progress to develop the diagnosis and treatment strategies for depression.

This study has some limitations. Although the high sensitivity and specificity of the microarrays provide a high level of confidence for the present findings, it can’t exclude the false negative rates due to the small sample sizes; thus, these findings needed to be experimentally verified in future studies. In addition, further studies are required to determine whether dysregulated inflammation is a response of the host to gut microbiota *per se* or a pathological change in depression caused by gut microbiota-dysbiosis.

## Conclusion

In summary, significant alterations have been identified in the composition of fecal microbiomes of patients with MDD. In the present study, the effects of depression-related gut microbiota on gene transcription in mice were analyzed to explore the pathological mechanisms of depression caused by gut microbiota dysbiosis, and found that dysregulation of the inflammatory response and neurodevelopment were the primary pathological changes. In addition, two microbial-regulated lncRNA–miRNA–mRNA ceRNA regulatory networks involved in depression-related neurodevelopment were constructed, one including two lncRNAs (4930417H01Rik and AI480526), one miRNA (mmu-miR-883b-3p) and two mRNAs (*Adcy1* and *Nr4a2*), and the other including six lncRNAs (5930412G12Rik, 6430628N08Rik, A530013C23Rik, A930007I19Rik, Gm15489 and Gm16251), one miRNA (mmu-miR-377-3p) and three mRNAs (*Six4*, *Stx16*, and *Ube3a*), and these molecules could be recognized as potential genetic and epigenetic biomarkers in microbial-associated depression. This study provides new clues for understanding the pathogenesis of gut microbiota-dysbiosis-induced depression and may act as a theoretical basis for the development of gut microbiota-based antidepressants.

## Data Availability Statement

The datasets presented in the present study can be found in GEO DataSets, and the GEO accession is GSE189234.

## Ethics Statement

The animal study was reviewed and approved by Ethics Committee of Chongqing Medical University.

## Author Contributions

PX and LL designed the work. HW, XC, YZ, and XR performed the experiments. WL and DY bred the germ-free mice. JP, LF, and SG analyzed the data. LL drafted the manuscript. YL, LZ, and PX revised the manuscript. All authors have read and approved the final manuscript.

## Conflict of Interest

The authors declare that the research was conducted in the absence of any commercial or financial relationships that could be construed as a potential conflict of interest.

## Publisher’s Note

All claims expressed in this article are solely those of the authors and do not necessarily represent those of their affiliated organizations, or those of the publisher, the editors and the reviewers. Any product that may be evaluated in this article, or claim that may be made by its manufacturer, is not guaranteed or endorsed by the publisher.

## Footnotes

1
http://www.omicsbean.cn/

2
http://www.ingenuity.com

3
https://string-db.org/
