# Supplementary material for: Integrative Analysis of Long Non-coding RNAs, Messenger RNAs, and MicroRNAs Indicates the Neurodevelopmental Dysfunction in the Hippocampus of Gut Microbiota-Dysbiosis Mice
Source: Front Mol Neurosci. 2022 Jan 11;14:745437. doi: 10.3389/fnmol.2021.745437 (PMC8787131; doi:10.3389/fnmol.2021.745437)
Supplement: Supplementary file 2 [file Image_1.pdf]

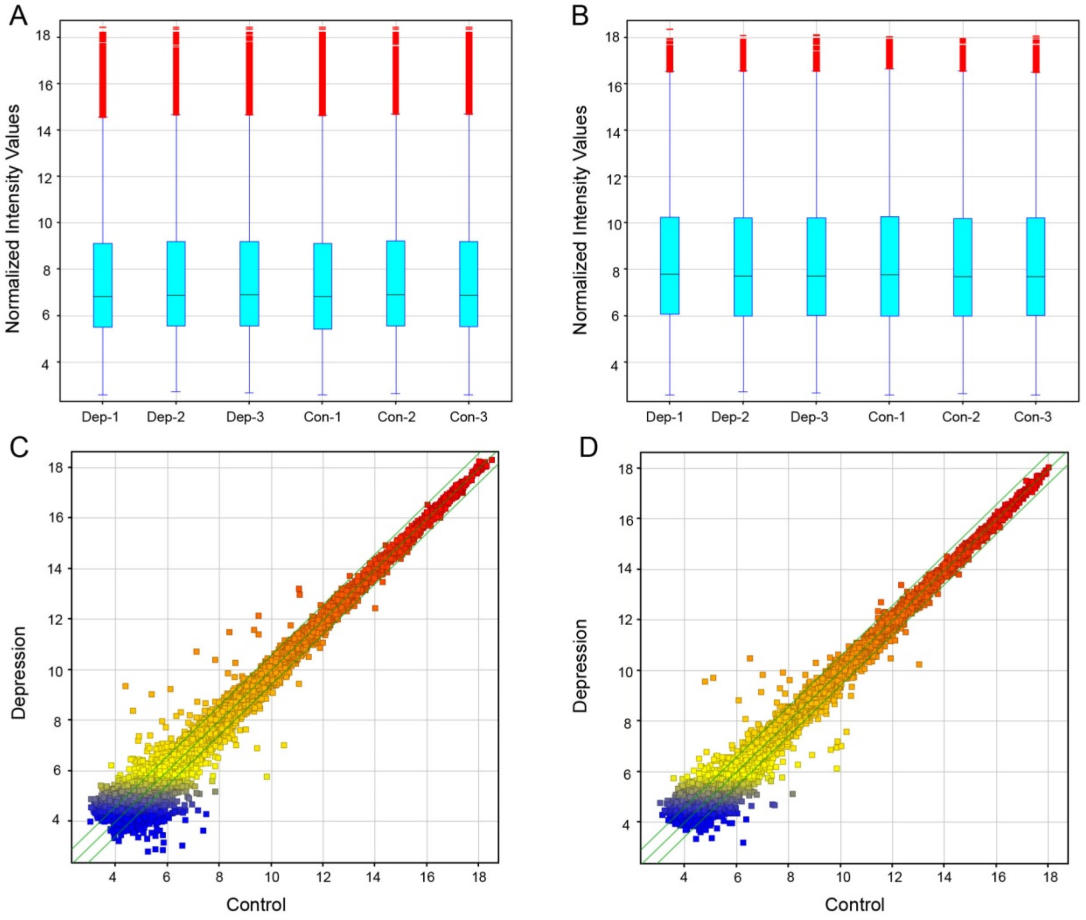

Figure S1. Quality assessment of lncRNA and mRNA data after filtering.

(A) Box-plot of lncRNAs showing that the distributions of normalized intensities among all tested samples are nearly the same. (B) Box-plot of mRNAs showing that the distributions of normalized intensities among all tested samples are nearly the same. (C) Scatter-plot assessing lncRNA expression variation and reproducibility between the two groups. (D) Scatter-plot assessing mRNA expression variation and reproducibility between the two groups.

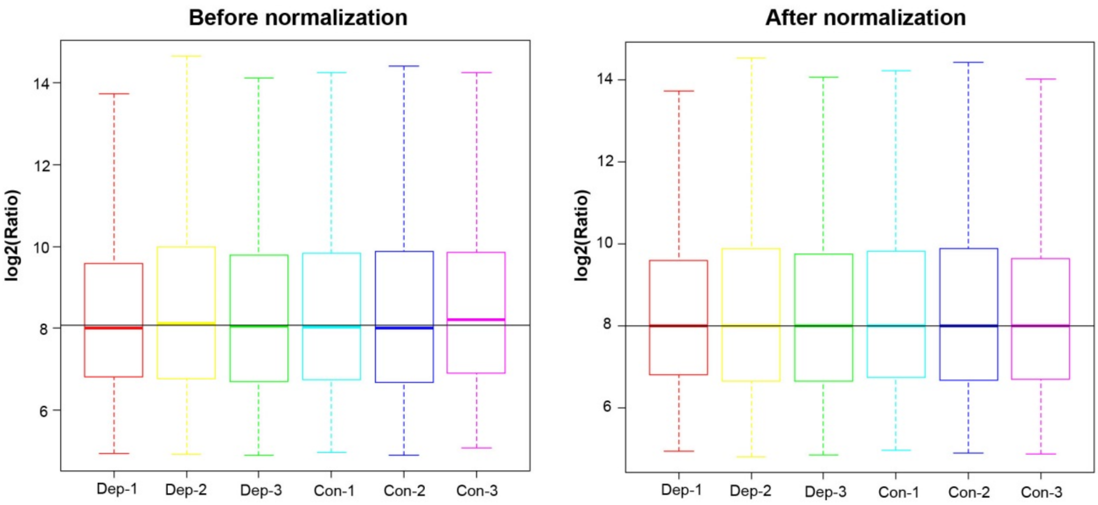

Figure S2. Box-plots of miRNAs visualizing the distribution of intensities for each sample before and after normalization.

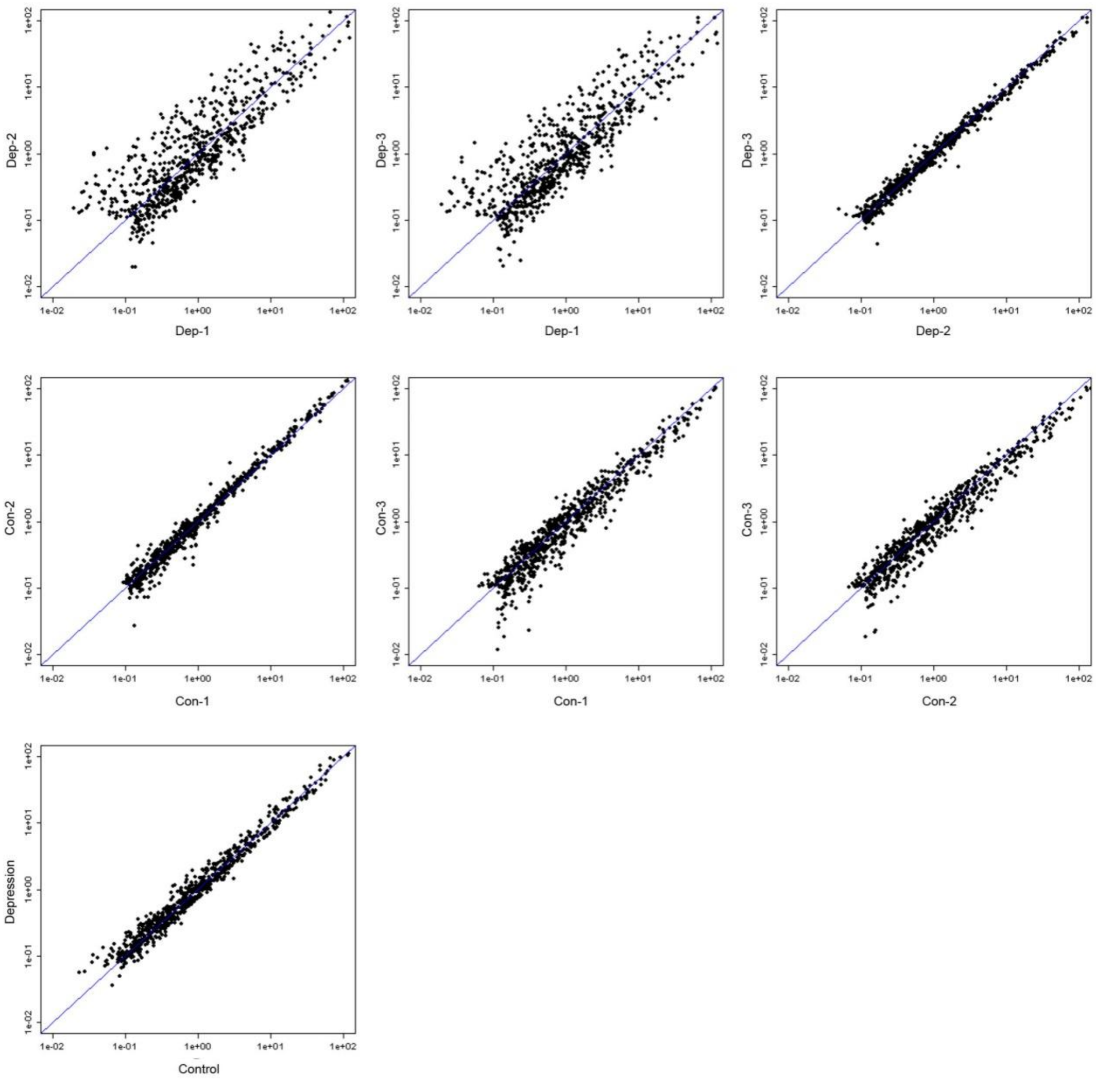

Figure S3. Scatter-plots assessing the correlation among replicate experiments.
